# Metabolomic effects of total flavone of *Abelmoschus manihot* (L.) medik. on patients with radiation-induced heart disease

**DOI:** 10.1038/s41598-025-24495-6

**Published:** 2025-11-19

**Authors:** Ruge Niu, Xiaolong Wang, Qi Jia, Wenqi Zhou, Long Zhang, Zhongchi Xu, Xin Lin

**Affiliations:** 1 Jiangsu Province Hospital of Chinese Medicine, Affiliated Hospital of Nanjing University of Chinese Medicine, #155 Hanzhong Road, Nanjing, 210029 People’s Republic of China; 2 College of Biotechnology and Pharmaceutical Engineering, Nanjing Tech University, #30 Puzhu Road, Nanjing, 211816 People’s Republic of China

**Keywords:** Gas chromatography–mass spectrometry, Radiotherapy, Radiation-induced heart disease, Metabolomics, Thoracic malignancy, TFA, Biochemistry, Cell biology, Diseases, Medical research

## Abstract

Radiation-induced heart disease (RIHD) is a severe complication of thoracic radiotherapy. Total flavone of *Abelmoschus Manihot* (L.) Medik. (TFA) demonstrates therapeutic potential on RIHD. However, its metabolic mechanisms remain elusive. This study aims to elucidate the change of serum metabolic profile of RIHD treated with TFA and identify potential metabolic pathways for mitigating irradiation damage. We randomly divided 100 RIHD patients into two groups, (1) TFA group: patients receiving TFA intervention (*n* = 50) and (2) non-TFA group: the other not receiving intervention (*n* = 50). The serum of patients was collected separately after the treatment. GC-MS metabolomics analysis employed to investigate differential metabolites in the serum of these patients. Multivariate (PCA/OPLS-DA) and univariate analyses identified differentially abundant metabolites (VIP > 1.0, *p* < 0.05, FC > 1.2) and enriched pathways. The non-TFA group exhibited profound metabolic disturbances characterized by mitochondrial dysfunction (depleted citrate with accumulated succinate/lactate), amino acid imbalance (elevated phenylalanine/tyrosine/tryptophan alongside reduced arginine and disrupted arginine-citrulline ratio), and lipotoxic stress (accumulated long-chain fatty acids including palmitic/arachidic acid with ketone body dysregulation). TFA intervention significantly reversed these perturbations: it restored citric acid cycle homeostasis through attenuated depletion of citrate and reduced succinate/lactate accumulation; rebalanced amino acid metabolism by lowering aromatic amino acids, elevating arginine levels to normalize the arginine/citrulline axis, and enhancing glycine/serine/threonine flux; and ameliorated lipid dysregulation via suppression of long-chain fatty acids and stabilization of ketone bodies. Pathway analysis confirmed that TFA can significantly regulate citric acid cycle, arginine biosynthesis, and fatty acid β-oxidation pathways. This study provides the first evidence that TFA counteracts RIHD metabolic pathology through coordinated mechanisms: TFA can repair mitochondrial dysfunction by restoring TCA cycle intermediates and reducing ROS generation. Meanwhile, TFA can reinforce redox defense by the inhibition of proteolysis-derived aromatic amino acids and the support of glutathione-precursor metabolism. Additionally, TFA can attenuate vascular injury by suppressing lipotoxicity while promoting endothelial NO synthesis.

## Introduction

Radiation-induced heart disease (RIHD) is a prevalent adverse effect in patients treated with radiotherapy for thoracic malignancies. The incidence and mortality rates of RIHD are rising as cancer survival rates continue to improve, making it one of the leading causes of morbidity and mortality among patients treated with radiotherapy^1^. The cardiovascular toxicity of ionizing radiation was first documented in atomic bomb survivors, with subsequent clinical observations revealing similar pathological manifestations in patients treated with radiotherapy^2,3^. Epidemiological studies have revealed that radiation exposure increases cardiovascular mortality by 17%–23% in atomic bomb survivors^4^, with similar trends observed in Chernobyl cleanup workers and medical radiotherapy cohorts^5,6^. A report from the United Nations Scientific Committee on the Effects of Atomic Radiation stated that all patients with cancer treated with radiotherapy lose an average of 0.6–0.7 years of life per Gy of radiation exposure, and that radiotherapy may increase the risk of circulatory diseases when the heart is exposed to high radiotherapy doses. However, the same report also noted that current scientific data are insufficient to determine the causal relationship between ionizing radiation at doses of < 1–2 Gy and cardiovascular diseases^1^. A meta-analysis encompassing 93 clinical studies provided a different view, suggesting that the relative risk of all cardiovascular diseases, including ischemic heart disease, is increased after radiation exposure, even in populations exposed to low radiation doses (maximum dose < 0.5 Gy) or low dose rates (< 5 mGy/hour)^4^. Other scholars have suggested that the rate of radiation-related mortality may be twice as high as currently estimated based on cancer endpoint estimates alone, supporting the association between mortality from cardiovascular diseases and exposure to low and moderate doses of ionizing radiation^7^.

*Abelmoschus manihot* (L.) Medik. is an annual herb native to East Asia whose flowers have been used to “clear heat, remove toxin and promote blood circulation” in traditional Chinese medicine (TCM) for more than 1600 years, which main active ingredients are flavonoids. Related research has shown that *Abelmoschus manihot* (L.) Medik. is rich in various bioactive compounds including hyperoside, isoquercitrin, myricetin, quercetin-3′-glucoside, quercetin and so on, which are believed to underpin its therapeutic effects^8,9^. Total flavones of *Abelmoschus manihot* (L.) Medik. (TFA) is a pharmaceutical formulation extracted from the flowers of *Abelmoschus manihot* (L.) Medik. under the trade name JiaHua Tablets. As a traditional Chinese medicine, it has been approved by the Jiangsu Provincial Food and Drug Administration of China (Approval No. Z04000511). In China, the medicinal use of *Abelmoschus manihot* (L.) Medik. was first documented in “Handbook of Prescriptions for Emergencies” by Hong Ge (in the Eastern Jin Dynasty, 317–420 AD) and later on collected in several medical books such as “Jiayou Materia Medica” (during the Jiayou reign of the Song Dynasty, 1056–1063), “Compendium of Materia Medica” (in the Ming Dynasty, 1368–1644) and “Dictionary of Chinese Pharmacy” (in 1934)^10^. TFA is now widely used in treating various ischemic cardiovascular and cerebrovascular diseases^11^. Related study has shown that TFA exhibits potent antioxidant and anti-inflammatory properties via modulating the Nrf2 signaling pathway to mitigate oxidative damage^12,13^. Another study found that TFA significantly reduces myocardial ischemia/reperfusion injury in rats^14^, while Wu et al. identified hyperoside-its primary active component-as protective against such injury by regulating mitochondrial dynamics, suppressing oxidative stress, and inhibiting apoptosis^15^. Furthermore, TFA has been shown to ameliorate diabetic nephropathy by inhibiting renal fibrosis and inflammation, and to protect against cerebral ischemia-reperfusion injury via anti-apoptotic mechanisms^16^. Additionally, emerging evidence suggests the extract may modulate ferroptosis to treat RIHD^17^. These multi-target and multi-dimensional pharmacological mechanisms collectively indicate that TFA possesses immense potential and broad application prospects in the prevention and treatment of radiation-induced heart disease (RIHD), making it a highly promising multi-target natural therapeutic agent.

Metabolomics is an emerging omics technology that has emerged in recent years. As the endpoint of omics cascades, metabolomic profiling directly reflects physiological perturbations and enables the identification of pathway-level changes with higher clinical translatability than genomic approaches. However, the mechanism by which TFA regulates metabolic pathways in RIHD remains unclear. Our study provides a novel perspective on this process, highlighting the potential of metabolomic profiling to uncover critical biochemical insights into therapeutic interventions.

**Fig. 1:**
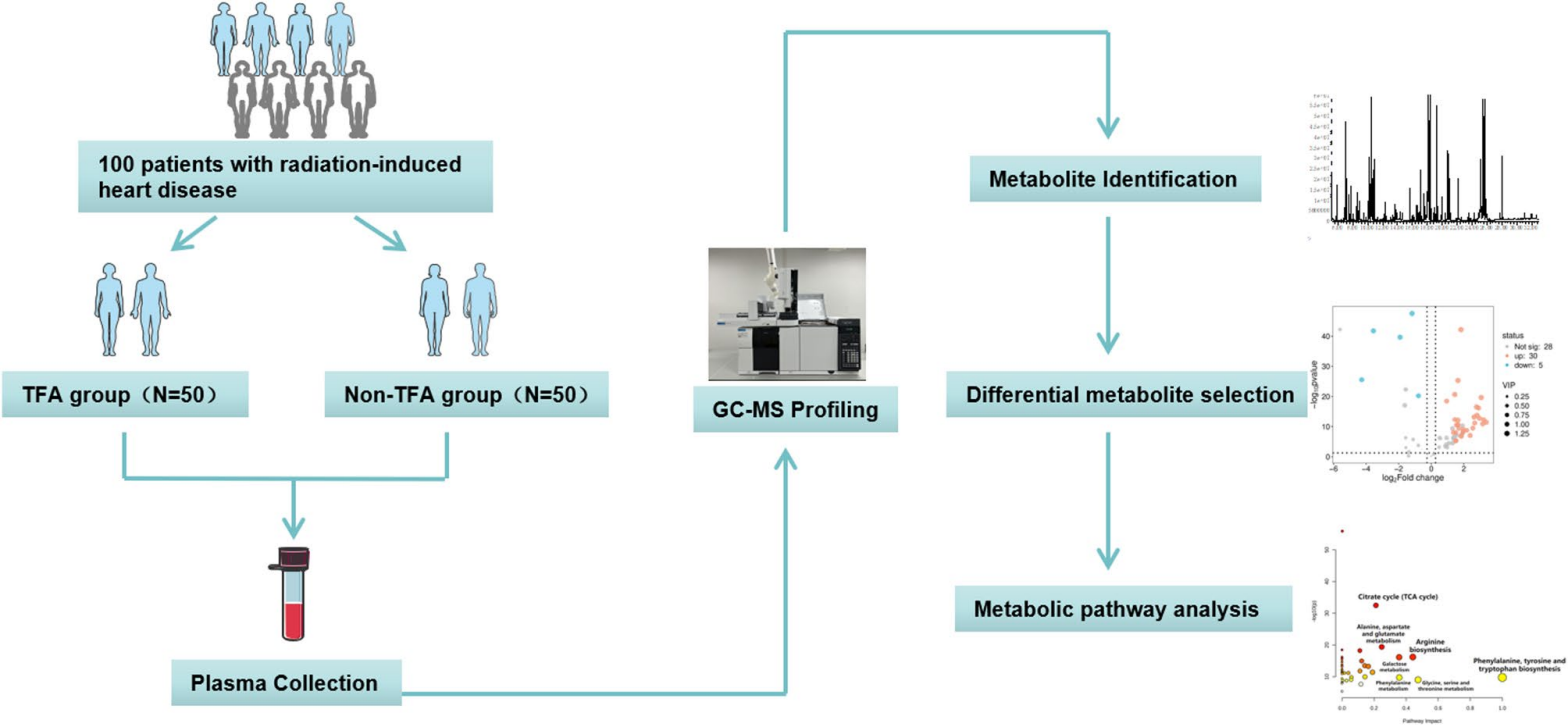
Study flowchart.

In this study, we performed a randomized controlled GC-MS metabolomics analysis comparing the seroprofiles of 100 patients with RIHD (50 patients who received TFA versus 50 untreated control patients). The therapeutic mechanism of TFA in RIHD was elucidated through a synthesis of non-targeted analysis and multivariate statistics, and the overall design is shown in Fig. 1.

## Materials and methods

### Chemicals and reagents

Myristic acid-d_27_ (internal standard) was purchased from CDN Isotopes (Pointe-Claire, Canada). Methyl myristate (external standard), methoxyamine, pyridine, trimethylchlorosilane (TMCS), heptane, and methanol were obtained from Sigma-Aldrich (Darmstadt, Germany). N-methyl-N-trimethyl-silyl-trifluoroacetamide (MSTFA) was obtained from Regis Technologies (Morton Grove, US).

### Clinical sample collection

All procedures performed in this study were carried out in accordance with the Code of Ethics of the World Medical Association (Declaration of Helsinki) for experiments involving humans and the ethical standards of the National Research Council. This study was approved by the Ethics Committee of Jiangsu Provincial Hospital of Traditional Chinese Medicine, Nanjing University of Chinese Medicine (approval number: 2023NL-261-02). Written informed consent was obtained from all participants prior to enrollment; for participants younger than 18 years, both parental/legal-guardian consent and the participant’s assent were obtained.

All enrolled participants (*n* = 100) were diagnosed with radiation-induced heart disease following thoracic radiotherapy for malignancies (predominantly Stage II–III non-small cell lung cancer and breast cancer according to AJCC 8th edition TNM staging) at Jiangsu Provincial Hospital of Traditional Chinese Medicine. Patients were randomly assigned to two equal cohorts (*n* = 50 per group): (1) TFA group: patients receiving conventional therapy plus TFA, and (2) non-TFA group: patients receiving conventional therapy alone. The TFA group received JiaHua Tablets at a dose of 4 tablets (equivalent to 1.8 g of crude drug) three times daily for 4 consecutive weeks. The major bioactive components of JiaHua Tablets include hyperoside (≥ 2.5 mg/tablet), isoquercitrin (≥ 1.5 mg/tablet), and myricetin (≥ 0.3 mg/tablet), as standardized by the manufacturer’s quality control protocols.

To ensure medication compliance, all participants in the TFA group received detailed medication education and were provided with medication diaries to record daily intake. Pill counts were conducted at each follow-up visit (every week). While plasma level monitoring of characteristic flavones would provide optimal compliance verification, this was not performed in the current study due to technical limitations. Instead, we relied on the combination of medication education, diary recording, and pill counting, which together provide reasonable assurance of medication adherence. Future studies would benefit from incorporating biochemical verification of compliance through plasma flavone measurements.

Patients with comorbidities other than thoracic tumors, including cardiovascular diseases (acute myocardial infarction, cardiogenic shock, pulmonary embolism, ventricular arrhythmia), severe uncontrolled infection, severe hepatic and renal insufficiency, digestive diseases, hematologic diseases, psychological problems, smoking, alcoholism, drug abuse, or hazardous occupations, were excluded.

RIHD was diagnosed according to the relevant criteria established by the American College of Cardiology and the European Society of Cardiology^18–20^, including radiation exposure, elevated markers of myocardial injury on laboratory tests (soluble suppression of tumorigenicity-2 > 50 ng/mL, cardiac troponin I > 50 pg/mL), and exclusion of other causes of heart disease.

Fasting venous blood (5 mL) was collected from all patients between 7 a.m. and 10 a.m., placed in a vacuum blood collection tube, and allowed to stand for 30–60 min before centrifugation (4 °C, 12,000 rpm, 15 min). After centrifugation, the supernatant was divided into cryovials and immediately frozen at − 80 °C for use in the metabolomic analysis.

### GC–MS data acquisition and metabolite identification

#### Sample preparation

First, 100 µL serum was aspirated, and 800 µL methanol containing myristic acid (30 µg/mL) was added to the serum. Samples were centrifuged at 14,000 rpm (4 °C for 10 min), 800 µL supernatant was collected, and 800 µL supernatant was dried in a centrifugal vacuum concentrator (Labconco, Missouri, US). The dried metabolites were re-dissolved and derivatized by adding 30 µL methoxyaminepyridine (15 mg/mL) for 16 h, followed by 30 µL MSTFA with 1% TMCS for silanization for an additional 1 h. Finally, 30 µL methyl myristate (30 µg/mL heptane solution) was added to each sample and, 65 µL was subjected to GC–MS analysis.

#### GC–MS analysis

An Agilent 7890 A GC system equipped with a 5975 C autosampler (Agilent Technologies, US) was employed for metabolomic analysis following standard procedures^21^. Samples (1 µL) were injected splitlessly onto the column using helium carrier gas flowing at 1 mL/min. The inlet and ion source temperatures were set to 250 °C. Chromatographic separation utilized a temperature gradient: 70 °C for 2 min, ramped at 10 °C/min to 300 °C, and held for 7 min, with a 5-min solvent delay. MS detection in scan mode (2.91 scans/sec) covered m/z 50–550. Putative compound identification was performed by comparing mass spectra and retention indices to entries in the Wiley and NIST 2.0 libraries.

### Statistical methods

Multivariate statistical analysis, including principal component analysis (PCA) and orthogonal partial least squares discriminant analysis (OPLS-DA), was conducted within SIMCA-P 14.1 software. The OPLS-DA model was evaluated through seven-fold cross-validation, and its quality and performance were assessed using Q2 (cum) for prediction accuracy, R2Y (cum) for fit assessment, cross-validation analysis of variance, and 200 iterative permutation tests to evaluate the risk of overfitting. Variable Importance in Projection (VIP) scores for metabolites were derived from this OPLS-DA model. Univariate statistical analyses were subsequently performed in MetaboAnalyst 5.0 (http://www.metaboanalyst.ca/MetaboAnalyst/) to compute fold change (FC) and *p*-values. Metabolites meeting the combined criteria of VIP > 1.0, either FC ≥ 1.2 or FC ≤ 0.83, and *p* < 0.05 were designated as statistically significant differential metabolites. These significant metabolites were then subjected to pathway enrichment analysis within MetaboAnalyst 5.0. Data are presented as mean ± standard deviation, and statistical significance was determined using Student’s t-test with *p* < 0.05^22^.

## Results

### Patients’ characteristics

As shown in Table 1, the data demonstrate well-balanced groups with no significant differences in age, body mass index (BMI), height, weight, heart rate, serum creatinine (Scr), urea, aspartate aminotransferase (AST), or alanine aminotransferase (ALT) levels between groups at baseline (all *p* > 0.05), ensuring that any subsequent metabolic differences can be attributed to the intervention rather than baseline characteristics.

**Table 1:** Baseline characteristics of study participants.

Parameters	TFA(*n* = 50)	non-TFA (*n* = 50)	*p*-value
Age (year)	66.50 ± 10.31	65.23 ± 11.53	0.638
BMI (kg/m^2^)	23.29 ± 3.43	23.67 ± 3.61	0.644
Height (cm)	164.36 ± 8.28	161.09 ± 7.90	0.074
Weight (KG)	63.59 ± 9.46	62.56 ± 10.68	0.670
Heart rate (bpm)	81.64 ± 13.24	77.35 ± 10.68	0.112
Scr (µmol/L)	72.37 ± 23.22	69.27 ± 23.35	0.578
Urea (mmol/L)	5.19 ± 1.25	6.05 ± 2.10	0.068
AST (U/L)	26.73 ± 18.05	25.32 ± 26.93	0.817
ALT (U/L)	22.27 ± 18.08	26.34 ± 34.77	0.597

### Metabolomic signature

**Fig. 2:**
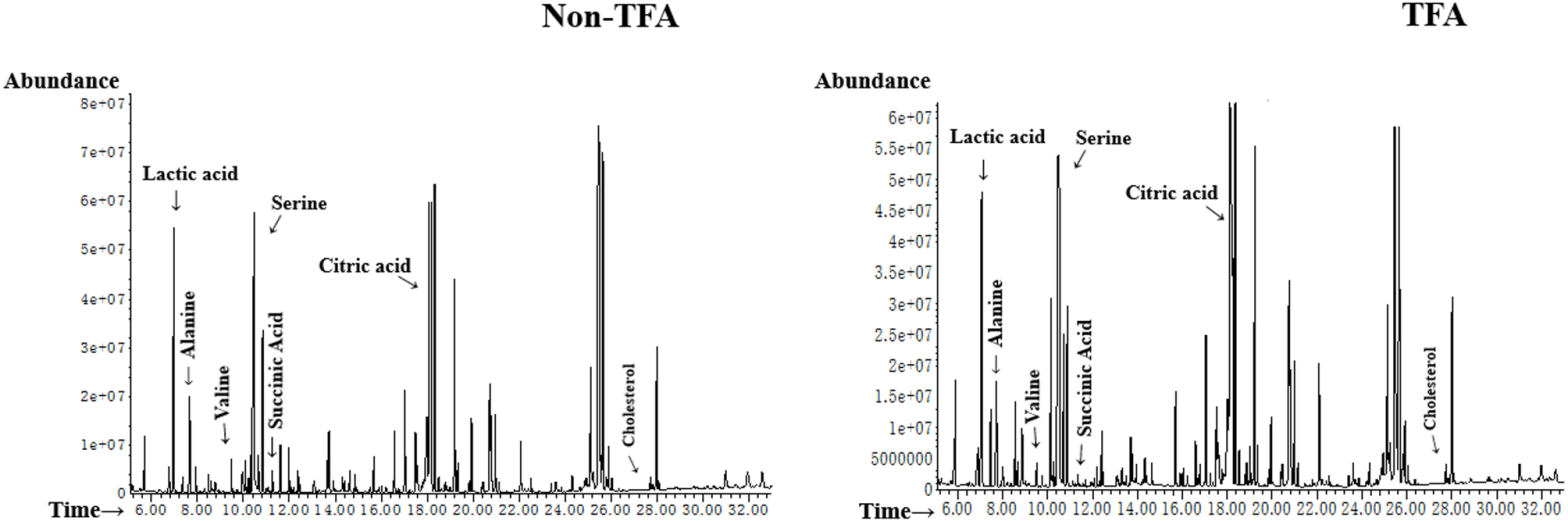
GC–MS total ion chromatogram of serum samples. Representative chromatograms show the metabolic profiles of non-TFA group and TFA group, with the x-axis representing retention time (min) and the y-axis indicating relative abundance. The complex pattern of peaks reflects the diverse metabolic landscape detectable by GC-MS analysis.

Representative GC–MS chromatograms of the serum samples from the two groups are shown in Fig. 2. We identified 63 compounds, including amino acids, organic acids, sugars, and fatty acids, using the NIST Library and the Wiley Library.

**Fig. 3:**
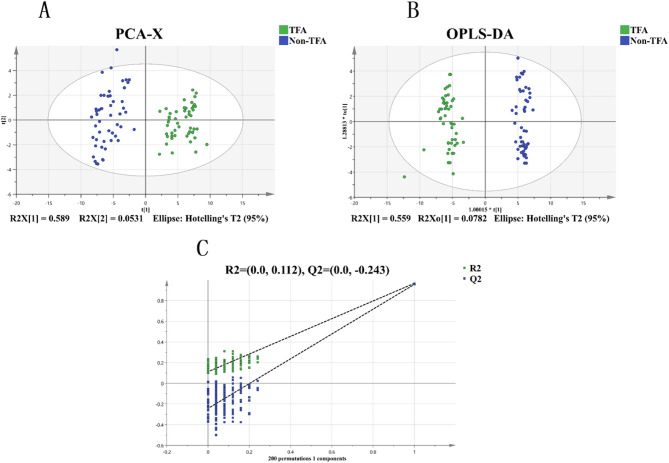
Multivariate analysis of serum metabolomic profiles. (**A**) PCA 2D score plot showing clustering patterns between TFA (green) and non-TFA (blue) groups. (**B**) OPLS-DA 2D score plot demonstrating clear separation between groups. (**C**) Alidation plot from 200 permutation tests showing the original model.

PCA was used to cluster the TFA and non-TFA groups into different regions (Fig. 3A), demonstrating clear separation between the two groups. OPLS-DA was performed to identify differences between the groups, and Fig. 3B shows that there was good separation between the two groups, indicating distinct serum small-molecule metabolite profiles. The OPLS-DA model was tested 200 times. The values of R² (cum) and Q² (cum) are represented on the y-axis, and the correlation coefficients between the original variable and the permutated y-variable are represented on the x-axis. The validation plot confirms the reliability of the initial OPLS-DA model, with the Q² (cum) regression line showing a negative intercept, indicating no overfitting and enabling confident screening of differential metabolites (Fig. 3C).

### Differential metabolite selection

To identify differential metabolites, we extracted VIP values from the OPLS-DA model. The VIP threshold was set at > 1.0, as this is a widely accepted criterion in metabolomic studies indicating metabolites with substantial contribution to group separation^23^. Higher VIP values indicated greater contribution of the metabolites to group segregation. With the Student’s t-test, *p* < 0.05 was considered statistically significant in the univariate analysis, while FC ≥ 1.2 or FC ≤ 0.83 was considered biologically significant.

**Fig. 4:**
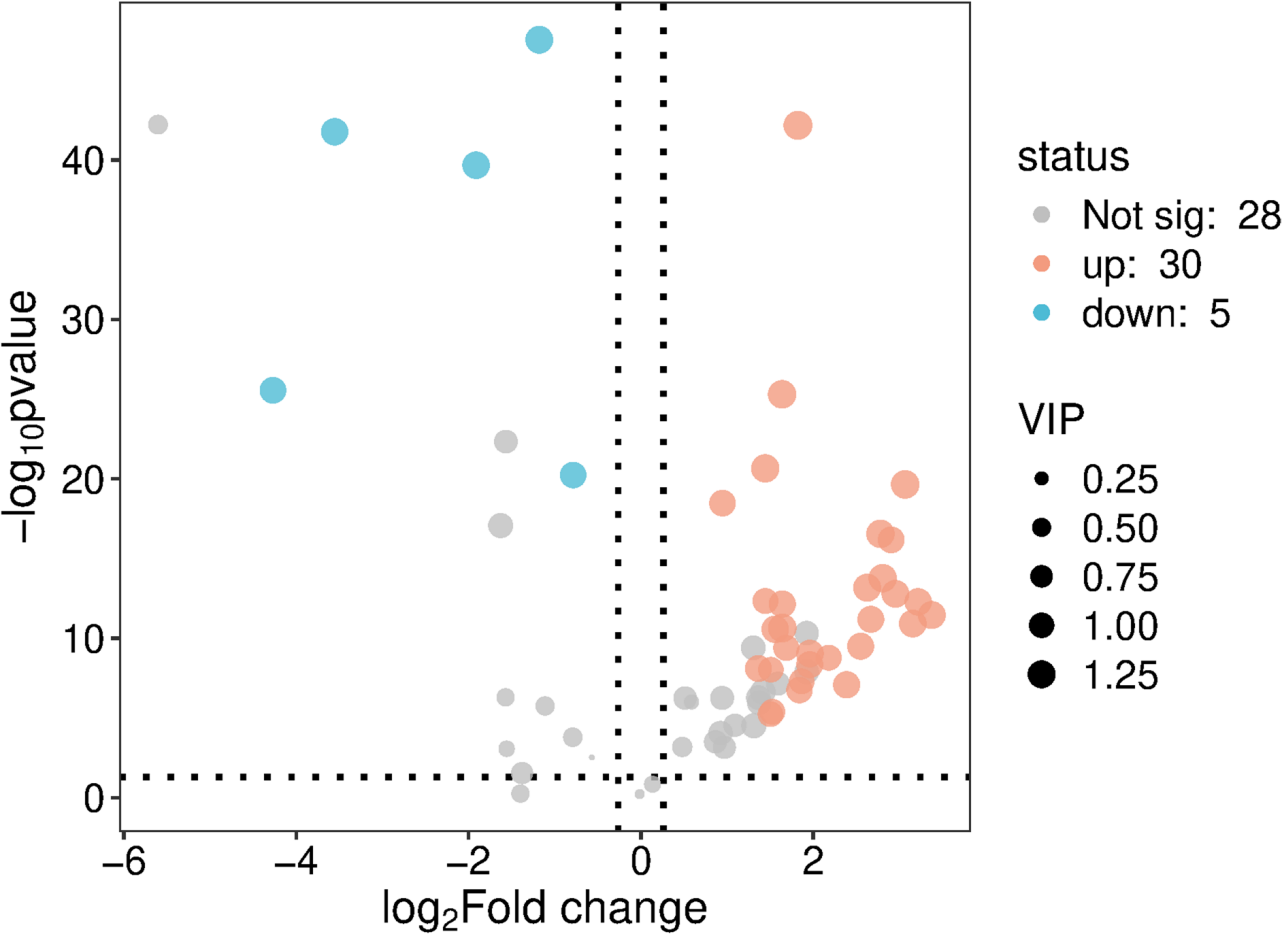
Volcano diagram of serum metabolites. Volcano plot displaying − log10 (p-value) versus log2(fold change) for all detected metabolites. Gray dots indicate no significant change (VIP < 1.0 or *p* > 0.05), red dots represent significantly upregulated metabolites in TFA group (VIP > 1.0, *p* < 0.05, FC ≥ 1.2), and blue dots indicate significantly downregulated metabolites (VIP > 1.0, *p* < 0.05, FC ≤ 0.83). The dashed horizontal line indicates *p* = 0.05 threshold, and vertical dashed lines indicate FC thresholds.

On the basis of the above analysis, 35 differential metabolites were identified according to the combination of VIP > 1.0, *p* < 0.05, FC ≥ 1.2, or FC ≤ 0.83, with 30 metabolites differentially upregulated and 5 differentially downregulated in the non-TFA group compared with the TFA group (Table 2). We combined statistical significance (p values) and FC to generate volcano plots to visually identify the data points with greater variability and statistical significance (Fig. 4).

**Table 2:** Potential biomarkers of radiation-induced heart disease were found by comparing the samples of the two groups.

Metabolite	Variation	VIP	*p*-value	FC
Lysine	↑	1.10944	3.33E-19	1.9282
Glucosamine	↑	1.14499	4.55E-09	3.8903
Cysteine	↑	1.13248	7.87E-09	2.5741
Rhamnose	↑	1.00267	5.82E-06	2.8358
Valine	↑	1.23029	1.63E-13	7.7554
Isoleucine	↑	1.28579	1.69E-14	6.9973
Tyrosine	↑	1.23758	3.49E-12	3.391
Threose	↑	1.31175	2.26E-11	3.1209
Hexadecanoic acid	↑	1.13759	6.45E-12	6.3597
Phenylalanine	↑	1.16638	8.26E-08	5.2349
Ketoadipic acid	↑	1.16244	7.13E-13	3.1199
α-Ketoglutarate	↓	1.18355	1.69E-42	0.0801
Arginine	↓	1.09916	5.73E-21	0.5799
Glycine	↑	1.0411	4.59E-13	2.7235
Cholesterol	↑	1.03605	1.65E-09	4.5371
Acetoacetic acid	↑	1.28289	2.20E-21	2.7196
Serine	↑	1.02413	4.08E-06	2.8797
Mannitol	↑	1.20632	9.00E-10	3.8965
Galactose	↑	1.19712	2.72E-11	2.9469
Glucopyranoside	↑	1.30035	4.97E-26	3.1156
Citrulline	↑	1.14821	3.16E-10	5.8614
Gamolenic Acid	↑	1.30628	2.24E-20	8.3868
Mannose	↑	1.24479	6.71E-14	6.174
Alanine	↑	1.00047	9.36E-09	2.8463
Palmitic acid	↑	1.09619	1.77E-07	3.5819
Glutamic acid	↑	1.22283	1.20E-11	8.9165
Proline	↑	1.28858	2.80E-17	6.8715
Arachidic acid	↑	1.33958	6.68E-43	3.5397
Ribose	↑	1.05081	4.03E-10	3.2215
Tryptophan	↑	1.01586	5.02E-08	3.6485
Fumaric acid	↓	1.19418	2.13E-40	0.2656
Citric acid	↓	1.23185	2.86E-48	0.4416
Lactic acid	↑	1.19549	5.21E-13	9.3101
Succinic Acid	↑	1.12868	6.69E-17	7.4945
Threonic acid	↓	1.12112	2.78E-26	0.0518

35 metabolites identified as significantly different between groups based on VIP > 1.0, *p* < 0.05, FC ≥ 1.2, or FC ≤ 0.83. Fold change values represent the ratio of metabolite concentrations in the control group relative to the TFA group, providing a standardized measure of differential abundance that facilitates cross-metabolite comparison and biomarker screening.

### Metabolic pathway analysis

**Fig. 5:**
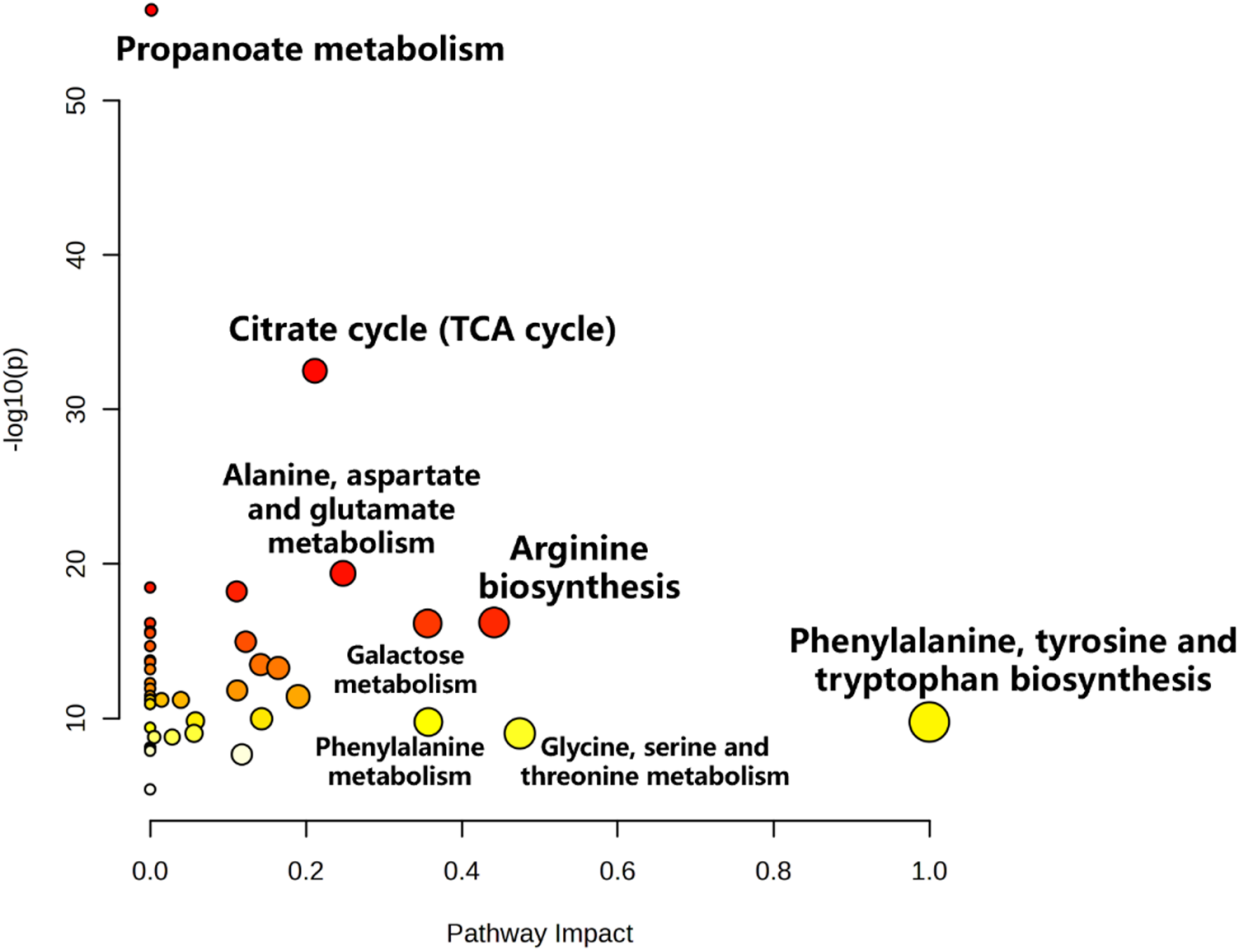
Overview of serum metabolite pathway analysis in non-TFA and TFA groups. Bubble plot showing significantly altered metabolic pathways between groups. The x-axis represents pathway impact value from topology analysis (based on betweenness centrality), and the y-axis shows -log10(*p*-value) from enrichment analysis. Bubble size corresponds to pathway impact value, and color intensity reflects statistical significance.

**Fig. 6:**
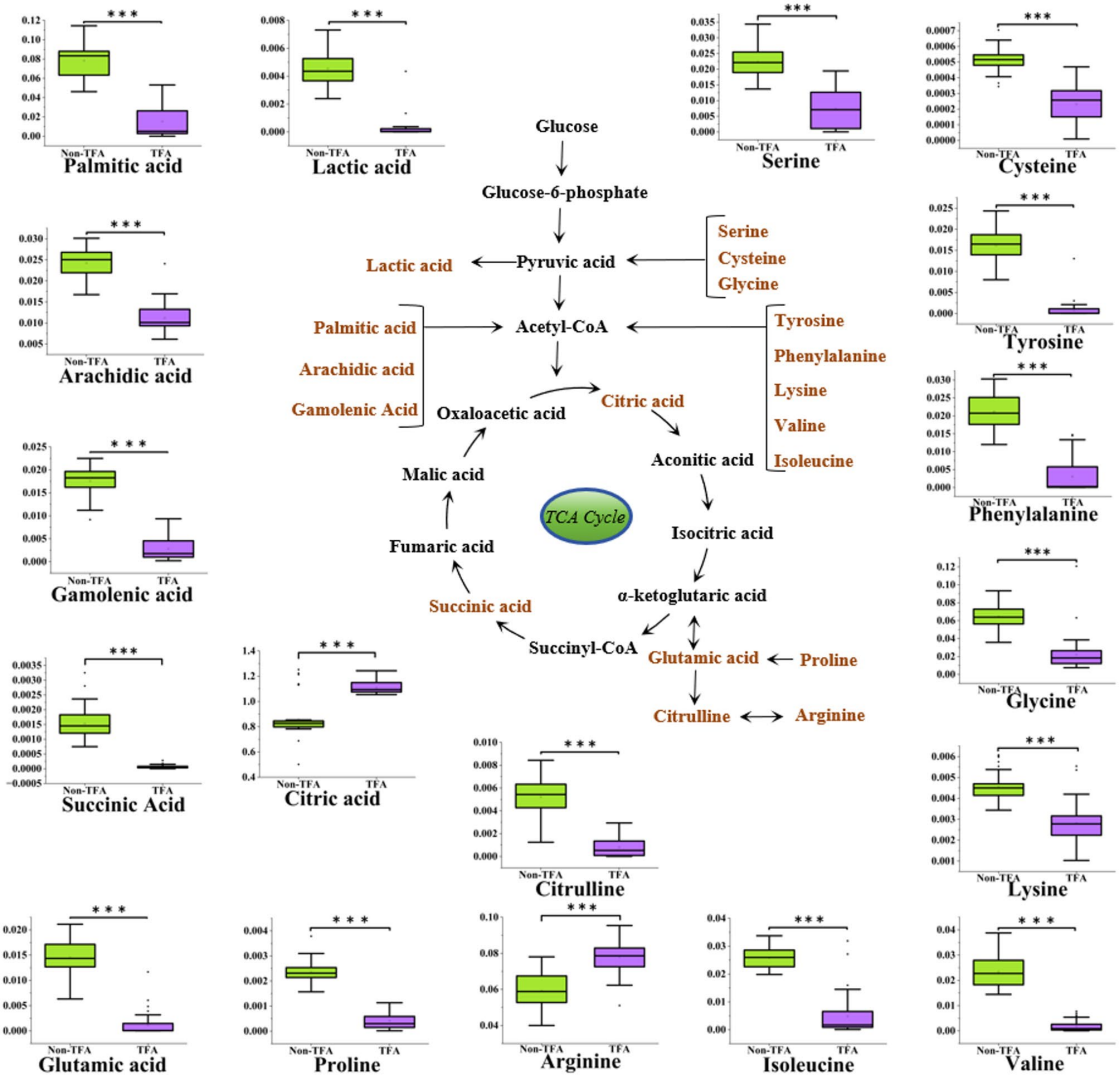
Schematic diagram of changes. Integrated metabolic network showing TFA-induced alterations in key pathways. Differential metabolites are indicated in brown font. The block plot shows the relative content of metabolites in the non-TFA group and the TFA group, ***p < 0.001.

Pathway enrichment analysis of differential metabolites was performed using MetaboAnalyst 5.0, revealing seven significantly perturbed pathways: phenylalanine/tyrosine/tryptophan biosynthesis, glycine/serine/threonine metabolism, arginine biosynthesis, phenylalanine metabolism, galactose metabolism, alanine/aspartate/glutamate metabolism, and citric acid cycle (Fig. 5). These TFA-modulated pathways collectively indicate mitochondrial dysfunction (TCA cycle arrest), impaired oxidative stress defense (arginine depletion and aromatic amino acid accumulation), and metabolic substrate dysregulation (amino acid/lipid shifts). To visualize TFA’s metabolic reprogramming effects, we constructed a serum metabolic network map contrasting intervention versus control groups (Fig. 6).

## Discussion

In this study, we performed the first GC–MS metabolomic analysis of serum specimens from RIHD patients to investigate the metabolic alterations associated with TFA intervention. Serum metabolic profiles of RIHD patients receiving TFA intervention (TFA group) were distinctly distinguished from those of RIHD patients without intervention (non-TFA group) by multivariate analysis, including PCA and OPLS-DA. Subsequent univariate analyses (FC and p values) identified 35 differential metabolites, including amino acids, fatty acids, and sugars, that characterize the metabolic response to TFA treatment. Pathway enrichment analysis suggested that TFA intervention influences carbohydrate, amino acid, and fatty acid metabolism pathways in RIHD.

Compared to the non-TFA group, the TFA group exhibited a metabolic profile suggesting a partial amelioration of mitochondrial dysfunction. While both groups showed perturbations, the TFA group demonstrated a significant attenuation in the depletion of citrate and α-ketoglutarate, and a reduction in the abnormal accumulation of succinate and lactate compared to the non-TFA group^24,25^. This pattern implies that TFA intervention may mitigate the specific radiation-induced damage to iron-sulfur cluster-dependent enzymes like aconitase and succinate dehydrogenase^26–28^, which drive the bidirectional arrest of the citric acid cycle and collapse of oxidative phosphorylation^29^. The less pronounced accumulation of lactate in the TFA group suggests a potential shift away from excessive anaerobic glycolysis, possibly due to improved energy status^30,31^. Furthermore, the observed changes might reflect TFA’s potential to reduce the burst of reactive oxygen species (ROS) caused by electron transport chain dysfunction^32^. This metabolic modulation by TFA shares similarities with interventions in ischemic cardiomyopathy^33^, supporting the concept of targeting energy metabolism in RIHD. By potentially alleviating this metabolic crisis and reducing ROS-mediated damage, TFA intervention might help mitigate downstream cardiomyocyte apoptosis and myocardial fibrosis^34^,thereby counteracting structural remodeling in RIHD.

TFA intervention significantly influenced amino acid metabolism profiles. The TFA group showed a marked reduction in the elevation of aromatic amino acids (phenylalanine, tyrosine, tryptophan) compared to the non-TFA group, suggesting that TFA may attenuate radiation-enhanced protein catabolism, potentially mediated by modulating the ubiquitin-proteasome system^35,36^. Importantly, TFA intervention appeared to partially restore the arginine-citrulline metabolic axis imbalance observed in the non-TFA group. Higher arginine levels and a more favorable arginine/citrulline ratio in the TFA group could promote nitric oxide (NO) biosynthesis^37^, counteracting the characteristic endothelial dysfunction of RIHD. This aligns with preclinical evidence of TFA components protecting endothelial function and NO synthase activity^38^. TFA also enhanced glycine/serine/threonine metabolism. This, coupled with the observed changes in aromatic amino acids, indicates that TFA may bolster a dual defense strategy: augmenting antioxidant capacity (potentially through supporting cysteine availability for glutathione synthesis) and promoting adaptive protein biosynthesis in response to radiation stress. Notably, TFA intervention mitigated the depletion of arginine and proline seen in the non-TFA group, which could help break the vicious cycle of disordered NO synthesis (exacerbating vascular injury) and abnormal collagen metabolism (driving fibrosis)^39^. The modulation of glutamic acid and aspartic acid levels by TFA warrants further investigation regarding its role in ammonia detoxification and energy metabolism adaptation^40,41^.

Serum lipid profiles revealed significant effects of TFA intervention. The coordinated elevations in long-chain fatty acids observed in the non-TFA group, indicative of disrupted β-oxidation and heightened risk of lipid peroxidation^42^, were significantly attenuated in the TFA group^43,44^. This suggests that TFA may promote more efficient fatty acid utilization and/or reduce lipid peroxidation, potentially lowering the risk for radiation-accelerated coronary artery stenosis and myocardial fibrosis^45^. The reduction in these pro-inflammatory precursors (eicosanoids, lipid mediators) in the TFA group also points towards an anti-inflammatory effect^46^. Furthermore, TFA intervention normalized the levels of ketoglutarate and acetoacetic acid compared to the non-TFA group, which could help interrupt the vicious cycle of enhanced fatty acid oxidation fueling mitochondrial ROS production and oxidative damage^47^. These findings support the potential of TFA to protect against radiation-induced vascular endothelial injury and atherosclerotic plaque formation by ameliorating lipid metabolic disorders^48,49^.

The observed metabolic improvements may be attributed to the rich flavone content of TFA, particularly hyperoside, isoquercitrin, and myricetin. These bioactive compounds are known to exert multifaceted protective effects through several mechanisms. First, flavones possess potent antioxidant properties, directly scavenging ROS and enhancing endogenous antioxidant defense systems by activating the Nrf2-ARE pathway^50,51^. Second, they exhibit anti-inflammatory effects by suppressing NF-κB activation and reducing pro-inflammatory cytokine production^52^. Third, flavones can modulate multiple signaling pathways involved in cell survival, including PI3K/Akt and MAPK pathways, thereby reducing apoptosis and promoting cell viability^53,54^. Fourth, they improve mitochondrial function by enhancing electron transport chain efficiency and reducing mitochondrial membrane permeability^55,56^. These mechanisms collectively contribute to the metabolic improvements observed in our study, particularly in mitigating oxidative stress, restoring energy metabolism, and reducing inflammatory responses in RIHD.

While the current study focused on metabolic changes, the observed improvements suggest potential clinical benefits of TFA intervention in RIHD patients. Previous clinical study on TFA has demonstrated significant benefits in various cardiovascular conditions, including reduced angina symptoms, improved exercise tolerance, and enhanced cardiac function^17^. Specifically for radiation-induced complications, traditional Chinese medicines containing similar flavones have shown efficacy in reducing radiation-induced inflammation, pain, and tissue fibrosis^57^. The metabolic normalization observed in our study, particularly in energy metabolism, oxidative stress markers, and lipid profiles, suggests that TFA intervention may translate to improved clinical outcomes such as reduced chest pain, decreased arrhythmia incidence, and better-preserved cardiac function.

This study has some limitations. The cross-sectional design makes it difficult to establish causality or definitively distinguish whether the observed metabolic changes in the TFA group are a direct driver of benefit or secondary to improved clinical status. Regarding treatment optimization, our selected regimen (4 tablets thrice daily for 4 weeks) demonstrated significant metabolic benefits, yet important questions remain. This dosage was based on: (1) Approved clinical guidelines (Jiangsu Provincial Drug Administration Approval No. Z04000511); (2) Previous pharmacokinetic studies showing effective plasma concentrations of key flavones (hyperoside > 50 ng/mL, isoquercitrin > 30 ng/mL) with this dosing regimen^58^. However, we recognize that optimal parameters may vary with disease severity. While 4 weeks sufficed for detectable metabolic improvement, advanced RIHD might require longer duration for complete metabolic normalization. Future studies should incorporate therapeutic drug monitoring of plasma flavone levels and employ adaptive trial designs to establish personalized dosing strategies.

## Conclusion

In conclusion, our GC-MS metabolomic analysis demonstrates that TFA intervention induces beneficial alterations in the serum metabolic profile of RIHD patients, primarily involving the TCA cycle, amino acid metabolism, and fatty acid metabolism. These effects are likely mediated by TFA flavonoids including hyperoside, isoquercitrin, and myricetin via their antioxidant, anti-inflammatory, and metabolic regulatory properties. Although further validation is needed to establish causal mechanisms, this study provides crucial insight into TFA’s therapeutic potential in RIHD.

## Data Availability

The datasets generated and/or analyzed during the current study are available from the corresponding author on reasonable request.

## Abbreviations

TFA: Total flavones of *Abelmoschus manihot* (L.) Medik.
RIHD: Radiation-induced heart disease
GC–MS: Gas chromatography–mass spectrometry
NIST: National institute of standards and technology
VIP: Variable importance in projection
PCA: Principal component analysis
OPLS-DA: Orthogonal partial least squares discriminant analysis
TMCS: Trimethylchlorosilane
MSTFA: N-methyl-N-trimethyl-silyl-trifluoroacetamide
BMI: Body mass index
AST: Aspartate aminotransferase
ALT: Alanine aminotransferase
